# Evaluating the Impact of Music & Memory’s Personalized Music and Tablet Engagement Program in Wisconsin Assisted Living Communities: Pilot Study

**DOI:** 10.2196/11599

**Published:** 2019-03-14

**Authors:** James H Ford II, Debby Dodds, Julie Hyland, Michael Potteiger

**Affiliations:** 1 School of Pharmacy University of Wisconsin–Madison Madison, WI United States; 2 Generation Connect York, PA United States; 3 Wisconsin Department of Health Servicess Madison, WI United States

**Keywords:** Music & Memory, assisted living facilities, quality of life, agitation, medication adherance, iPod, iPad, implementation science, patient participation

## Abstract

**Background:**

Individuals with Alzheimer disease or related dementia represent a significant and growing segment of the older adult (aged 65 years and above) population. In addition to physical health concerns, including comorbid medical conditions, these individuals often exhibit behavioral and psychological symptoms of dementia (BPSD). The presence of BPSD in long-term care residential facilities can disrupt resident’s care and impact staff. Nonpharmacological interventions such as personalized music and tablet engagement maintain cognitive function, improve quality of life (QOL), and mitigate BPSD for older adults with dementia. Evidence of the impact of such interventions in assisted living communities (ALCs) is needed for widespread adoption and sustainment of these technologies.

**Objective:**

The aim of this study was to assess the impact of Music & Memory’s personalized music and tablet engagement (PMATE) program on QOL, agitation, and medication use for residents living in 6 Wisconsin ALCs.

**Methods:**

The data collected were on the utilization of iPods and iPads by the residents. Residents’ outcomes were assessed using the Pittsburgh Agitation Scale, the Quality of Life in Late Stage Dementia scale, and self-reported medication use. A mixed-methods approach was utilized to examine the impact of the PMATE program on these outcomes. Descriptive statistics were calculated. A paired *t* test explored changes in residents’ QOL. A 1-way analysis of variance was utilized to examine changes in resident’s agitation and QOL based on the resident’s utilization of the PMATE program. Qualitative interviews were conducted with the individuals responsible for PMATE implementation in the ALC. Residents excluded from the analysis were those who passed away, were discharged, or refused to participate.

**Results:**

A total of 5 apps, based on average times used by residents, were identified. In all, 4 of the 5 apps were rated as being useful to promote residents’ engagement. PMATE utilization was not associated with changes in the residents’ agitation levels or antipsychotic medication use over time. Over a 3-month period, the change in residents’ QOL was significant (*P*=.047), and the differences across ALCs were also significant (*F*_25_=3.76, *P*=.02). High utilizers of the PMATE program (>2500 min over 3 months) showed greater improvements in QOL as compared with low utilizers (a change of −5.90 points vs an increase of 0.43 points). The difference was significant (*P*=.03). Similar significant findings were found between the high- and midutilizers.

**Conclusions:**

The study is one of the first to explore the impact of Music & Memory’s PMATE program on residents living in ALCs. Findings suggest that higher utilization over time improves residents’ QOL. However, a more comprehensive study with improved data collection efforts across multiple ALCs is needed to confirm these preliminary findings.

## Introduction

### Background

Individuals with Alzheimer’s disease or related dementia (ADRD) represent a significant and growing segment of the older adult (aged 65 years and above) population. In 2013, approximately 5 million older adults were diagnosed with ADRD with an estimated US $214 billion spent on medical office visits, medications, or formal/informal caregiving [1,2]. Long-range forecasts project that the number of older adults aged 65 years and above with ADRD will almost triple to 14 million by 2050 and health care expenditures will more than double to US $511 billion by 2040 [1,3,4]. This dramatic rise will place a major burden on long-term care facilities such as nursing homes (NHs) and assisted living communities (ALCs). Studies suggest that between 42% and 67% of residents living in ALCs have some form of moderate-to-severe cognitive impairment [5-7]. The state of Wisconsin is experiencing similar trends. In 2010, Wisconsin had almost 120,000 residents with ADRD. With a capacity for approximately 55,000 residents to reside in Wisconsin ALCs, national trends [1] would suggest that at least 15,500 of these individuals, or 28% of ALC residents, have some form of dementia. In addition, 48.7% of licensed Wisconsin ALCs provide care to individuals with ADRD [8].

### Dementia Complications

Aggression, agitation, anxiety, or sundown syndrome are examples of behavioral and psychological symptoms of dementia (BPSD). These symptoms are frequently exhibited by individuals with moderate-to-severe dementia [5,9-12], can disrupt residents’ care, and negatively impact staff and residents’ quality of life (QOL). Although medications can control the physical aspects of BPSD, the medications may impact residents’ health, diminish a resident’s ability to participate in life events, and accelerate the disease [13-15]. However, there is currently an emphasis on person-centered care [16] or care that accounts for individual preferences. Some person-centered interventions for dementia include cognitive or motion-oriented (eg, reminiscence therapy), sensory stimulation (eg, visual or music therapy), and behavior management techniques [17]. These nonpharmacological interventions maintain cognitive function, mitigate individual behavior issues for older adults with dementia, improve residents’ agitation levels, improve QOL, and reduce medication use for nursing home residents with dementia [18-21].

### Music Interventions

Music therapy is a type of nonpharmacological intervention involving individual engagement through active or passive listening [22,23]. The impact on residents’ outcomes is mixed. For example, a group music therapy intervention (ie, listening to music) has been shown to have no effect on changes in aggressive behaviors such as agitation and anxiety [24-28]. Other studies suggest that music therapy, depending on its focus (active vs passive), delivery mechanism (individual vs group session), and frequency (weekly vs biweekly), improves residents’ BPSD including aggression, agitation, anxiety, and sundown symptoms, as well as QOL [27-36]. Individual music therapy sessions reduce agitation, disruptiveness, combativeness, and elopement, as well as the use of restraints and medications in individuals with dementia [37-39]. However, these studies were conducted in NHs or hospitals. Therefore, it is unclear if a personalized music intervention would improve BPSD or QOL for individuals with dementia living in ALCs. This study evaluated personalized music and tablet engagement (PMATE) programs in Wisconsin ALCs.

## Objectives

### Music & Memory Overview

The Wisconsin Department of Health Services (DHS) introduced Music & Memory to Wisconsin NHs in 2013 after the staff attended a conference where they viewed a video clip featuring Henry coming alive while listening to his personalized music [40]. Henry, the star of the 2014 Sundance Award—winning documentary film *Alive Inside, A Story of Music & Memory* —was among the first individuals Dan Cohen, MSW, Executive Director of Music & Memory [41], assisted in reconnecting with his personhood. The film highlights the power of individualized music to unlock the minds of those living with ADRD. The film follows Cohen in his efforts to “demonstrate music’s ability to combat memory loss and restore a deep sense of self to those suffering from it” [42,43]. The mission of Music & Memory is to bring “personalized music into the lives of the elderly or infirm through digital music technology, vastly improving quality of life.”

Music & Memory is a personalized music program, not music therapy. Individual care professionals or a team of caregivers is trained to create powerful personalized music playlists for each individual participant. The personalized music is provided to the participant through the use of iPods and other related digital audio devices. The personalized music enables individuals living with ADRD, as well as those with other cognitive and physical challenges, to reconnect with the world through music-triggered memories.

Wisconsin DHS was the first to adopt Music & Memory as a statewide initiative, with 25 states following suit. At the time of adoption, Music & Memory was in less than 700 NHs worldwide. As of 2018, more than 5000 organizations have adopted Music & Memory, with more than 400 organizations in Wisconsin [41]. Expansion is not just measured by the number of organizations who have adopted the program but also by the adoption in a wide variety of environments including ALCs and NHs as well as in the community [41].

In addition to the use of iPods, Music & Memory has introduced the use of iPads or tablets to further personalize the engagement of individuals with dementia. For this study, the term *engage* or *engagement* with regard to iPads or tablets refers to residents choosing to participate or not participate in iPad or tablet activities. These software app–driven activities include playing familiar games, reminiscing about life stories with music, viewing Web-based images or using Google Earth, dabbling in art, and reactivating lifelong learning interests.

iPads or tablets enhance residents’ emotions through the use of multisensory activities. The availability of a wide range of software and apps, and internet access, allows for tailoring to residents’ interests or hobbies, resulting in more meaningful, pleasurable activities for the residents. YouTube, entertainment, family photographs, games, Alzheimer’s apps, music videos, and Skype, for example, help reduce residents’ boredom and isolation while enabling greater independence, productivity, connection, and socialization [44]. Also, one study found that iPads increase caregiver confidence and their ability to engage socially and improve their personal life and health [45].

### Music & Memory Impact

Further studies indicate that Music & Memory’s personalized music program can reduce disruptive behaviors, major depressive episodes, residents’ behavioral disturbances, and the use of antipsychotic or anxiolytic medications; improve residents’ moods and reduce disruptive behaviors; or enhance swallowing in individuals with advanced dementia [46-48]. For example, an implementation in 2 memory care units within NYC Health + Hospitals, involving more than 100 patients, showed a reduction in the use of antipsychotic medications, physical altercations, and falls for residents who participated in the Music & Memory program [46]. However, results were mixed from several Wisconsin NH Music & Memory pilot projects targeting residents (eg, agitation and medication use) or caregivers (eg, job satisfaction and distress) [49-51]. These early implementations of Music & Memory lacked the robust support and training systems of today, including an online care community that promotes collaboration, continuous Web-based training for staff turnover and program expansion, and webinars to help NHs with implementation for specific populations.

Despite these findings, a more effective evaluation of nonpharmacological interventions, such as PMATE in ALCs, is needed [52]. Furthermore, it has been suggested that the intervention focuses not only on the personalization of the music (eg, linkage to personal history) but research should also explore the impact of music on individual resident and facility outcomes [53]. This study addresses this gap and reports on the implementation of Music & Memory’s PMATE program in 6 Wisconsin ALCs and its impact on individual residents’ outcomes.

## Methods

### Study Setting

This pilot study evaluated the impact of the PMATE program on residents’ agitation and QOL in a convenient sample of ALCs in Wisconsin. Eligibility criteria included being an ALC in good standing with the Wisconsin Coalition for Collaborative Excellence in Assisted Living (WCCEAL) [54], having a primary population of individuals with ADRD, 25 beds or less, and a minimum of 4 residents on antipsychotic or antianxiety medication. An application (see Multimedia Appendix 1) was developed to assist with recruitment, which described the study including eligibility criteria. Upon completion of the application, interested ALCs agreed to implement Music & Memory as trained and to participate in the study evaluation. The 4 Wisconsin assisted living associations helped in recruiting ALCs that were also WCCEAL members. The association staff selected 6 ALCs (see Multimedia Appendix 2 for attributes) from the 14 applications received to participate in this initiative. After completing Music & Memory’s PMATE training (see the training section for details), each ALC was provided with the equipment needed to implement the program. The equipment included a US $100 iTunes gift card, 1 external speaker, 1 headphone splitter, an average of 6 iPod Shuffles, headphones, and alternating current adapters for resident or tenant use. In addition, each ALC received an iPad Mini and iPad Pro. The iPad Pro, with a 12.9-inch retina screen, was provided for ease of residents’ viewing. The iPad Mini was intended for staff training and familiarity and was not intended for use with residents. To help provide focus for the tablet sessions and to help promote positive outcomes, the apps selected and loaded on the iPads were centered on traditional activity-based subjects, such as reminiscing, that are both beneficial and engaging for people with cognitive impairment. The process of choosing the apps for this study was largely based on the research for, and results of, two community memory loss iPad programs [55,56], created by author DD.

These initial iPad memory loss programs used apps from 4 basic types of engagement activities—reminiscing, music, images, and games—which have been conceptualized differently for this study. Music & Memory added the app categories of lifelong learning and relaxation, and music was not considered as an app category owing to the study’s use of personalized music with iPods. The final tablet engagement categories included apps from relaxation, lifelong learning, life stories, and games, as defined in the Data Collection section.

All apps loaded onto the iPad were available in the public domain. The most important requirement for successful app selection within the categories was deemed to be simplicity. However, simplicity is not the goal of most app developers. Bells and whistles, such as timers on games, pop-up ads, flashy elements to entice use, and gamification in general, are not helpful for people with memory loss and can, in fact, detract from the apps’ effectiveness. In addition, the childish nature of simple apps can often infantilize the older users. For example, while looking for an easy crossword puzzle, it was discovered that the majority of simple puzzles were made for children with character sounds and rewards; see Multimedia Appendix 3 for a description of the apps included on the iPad.

### Music & Memory Staff Training

Participating ALCs designated one person from the staff to serve as the project lead and another to serve as an alternate. The staff received training (three 90-min live webinars) on how to implement Music & Memory’s personalized music program within their facility as well as 3 60-min live webinar trainings on how to use the iPad and the associated apps. Communities were encouraged to have all interested staff attend the training, as greater success is evident in organizations when multiple staff members receive training and are involved in the day-to-day operations of the program. Communities became certified as a Music & Memory organization upon completion of the training. The value of the training and equipment, provided at no cost to each ALC, was over US $1500. Intervention checks were not completed outside of the initial Music & Memory training or the evaluation. Due to high staff turnover, one ALC received an in-person visit from Music & Memory’s dedicated staff member in the state. The ALC staff was encouraged to request additional support, as needed, which included access to Music & Memory’s dedicated staff members.

### Data Collection

The data collection in the study included residents’ outcomes related to agitation, QOL and medication use, utilization of iPods to listen to music (frequency and length of time), app utilization on the iPad (frequency, time, and impact on the resident), and information on antipsychotic medications. In addition, phone interviews were conducted with the project lead from each participating ALC to understand how integrating PMATE into daily care impacted staff interactions with residents. Each data collection item is described below.

The Pittsburgh Agitation Scale (PAS) allows an observer to rate the severity of agitation related to dementia within individuals [57]. The PAS examines 4 general behavior groups: (1) aberrant vocalizations; (2) motor agitation; (3) aggressiveness; and (4) resisting care related to washing, dressing, eating, and medications. Raters used a 4-point scale when assessing residents’ behaviors over a 7-day period. Application of the PAS in a geropsychiatric unit (ICC=.82) and NH (ICC=.93) showed high inter-rater reliability [57]. The PAS also inquires about hours of sleep for the residents during the observation period. In this study, the ALC staff were asked to conduct a weekly assessment (Monday to Friday) on each resident, and based on their observations, the residents’ level of daily agitation was rated. In addition, the staff recorded hours of sleep during the rating period and whether physical restraints, PRN medications, Music & Memory, or other interventions were utilized to control a resident’s agitation. PAS data were collected at 4 time periods in 2016: April 4 to April 8, May 2 to May 6, June 6 to June 10, and July 4 to July 8.

The Quality of Life in Late Stage Dementia (QUALID) is an 11-item scale that measures the QOL for an individual with late-stage dementia [58]. A professional caregiver who had regular resident contact and was familiar with the resident’s general behavior completed the QUALID scale. The QUALID scale has high internal consistency reliability (alpha=.77), test-retest reliability (ICC=.81), and inter-rater reliability (ICC=.83) [58]. The individual completing the QUALID scale must have spent a significant portion of 3 of the last 7 days with the resident to accurately rate the items on the scale. To calculate a QUALID scale score, each item is scored on a 1 to 5 scale with the sum of the scores ranging from 11 to 55 points to represent the residents’ overall QOL. Lower scores represent a better QOL. For this study, the ALC staff assessed residents’ QOL at baseline on or around April 11 or 12, 2016, reflecting the residents’ behavior during the week of April 3 to April 8, 2016, and again post intervention on or around July 12 or 13, 2016, related to residents’ behavior during the week of June 4 to July 8, 2016.

For each participating resident, the ALC staff was asked to track residents’ use including dose and frequency of antipsychotic medications from March through June 2016. The ALC staff also recorded iPod and iPad app utilization (date and time) and ranked the usefulness of the app in promoting residents’ engagement using a 4-point scale (4=Very Useful, 3=Somewhat Useful, 2=Not Very Useful, and 1=Not at all useful). As a condition of study participation, residents’ demographic information was not collected; therefore, a resident’s profile could not be established.

The research team trained the staff from the participating ALCs on how to collect and report the data and provided a project binder to record the outcomes for each resident who participated in the pilot. The pilot timeline is shown in Multimedia Appendix 4. An overview of the data collection process is shown in Multimedia Appendix 5.

### Data Analysis

Descriptive statistics related to residents’ agitation, QOL, and change over time were calculated. No resident characteristics were collected as a part of this pilot study per the agreement with the assisted living associations.

In addition, iPod and iPad utilization (time and count) were calculated across the sample and by participating ALCs. The iPad app use statistics assessed the total number of times an app was used; the total number of residents using an app as well as the maximum number of times a specific resident used an app; the average number of times an app was used across all residents; and the percent of residents utilizing a given app. Quartiles based on total iPod and combined iPod and iPad use in minutes were determined. Statistics on the average usefulness of each iPad app were also determined.

A one-way ANOVA was utilized to examine changes in residents’ agitation as well as the 4 behavior groups, scored by month when the intervention was the PMATE (0=No, 1=Yes) and if changes in the overall agitation score was related to total iPod or iPod and iPad utilization by quartile. A stepwise regression explored if the residents’ agitation score from the previous day, hours of sleep during the observation period, and the use of the PMATE program could predict the current agitation score.

A paired sample *t* test determined if residents’ QOL scores changed significantly over time. Residents were grouped based on their total PMATE utilization into 3 categories: <1000 min; between 1000 and 2500 min; and >2500 min of total use. A one-way ANOVA was utilized to determine if pre- and post-QOL scores differed by ALC and to explore changes in QOL scores by ALC and total PMATE utilization in minutes. Missing values for the pre-QOL scores were imputed, and a sensitivity analysis based on a sample of 29 matched pairs was performed to verify results.

Residents who passed away, were discharged, or refused to participate (n=6) were excluded from the analysis. Interviews were conducted with all 6 participating ALCs about their experiences with the PMATE program in their community. The analysis focused on the impact of the intervention on ALC residents and its impact on staff interactions. The study has been approved by the Health Sciences Minimal Risk Institutional Review Board at the University of Wisconsin–Madison (2016-0835).

## Results

Data collection notebooks were returned by 5 of the 6 ALCs and contained data from 35 residents. Table 1 shows the utilization of the iPod and iPad by residents within an ALC and the total across all residents in the ALC.

**Figure 1:**
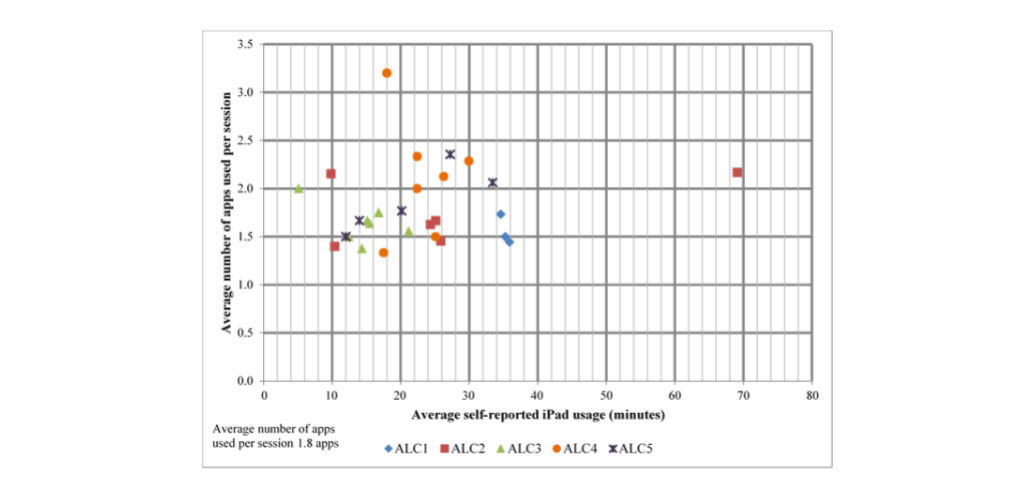
Assisted living community resident iPad app use by average apps used per session versus self-reported iPad use in minutes.

The frequency of iPad app utilization and usefulness in promoting residents’ engagement was determined for all residents and for all residents excluding those who passed away, were discharged, or refused to participate. The resulting information is shown in Multimedia Appendix 6. The top 5 apps (based on average times used by resident) in both groups were (1) Puzzable, (2) Colorfy, (3) Ted Talks, (4) Image Search, and (5) Words-Osmo. Across the 2 groups, 4 of the top 5 apps in terms of usefulness to promote residents’ engagement were also the most frequently utilized (Image Search, Garage Band, Take a Break, and Ted Talks). Across all residents, Adobe Voice was rated more useful as compared with Puzzable for the exclusionary sample of residents. Figure 1 shows the average number of apps used per session versus the average minutes of iPad usage by residents within the ALC. Across the sample, residents utilized an average of 1.8 apps per session for approximately 23 min. Of the other apps utilized, 57 were not identified in the workbook. Of those identified, 26 uses were for Baby Bath, 2 for Music (unidentified), and 1 each for a Nature app, Talking Ben, YouTube songs, and YouTube and Photo.

Residents’ agitation scores (total and by individual behavior group) decreased over time. However, no significant differences in PMATE utilization to address residents’ agitation were found (results not shown). Similar nonsignificant findings were found from the regression analysis as well as the comparison with the change in overall agitation score by total utilization, measured in minutes, of the iPod as well as the iPod and iPad combined.

**Figure 2:**
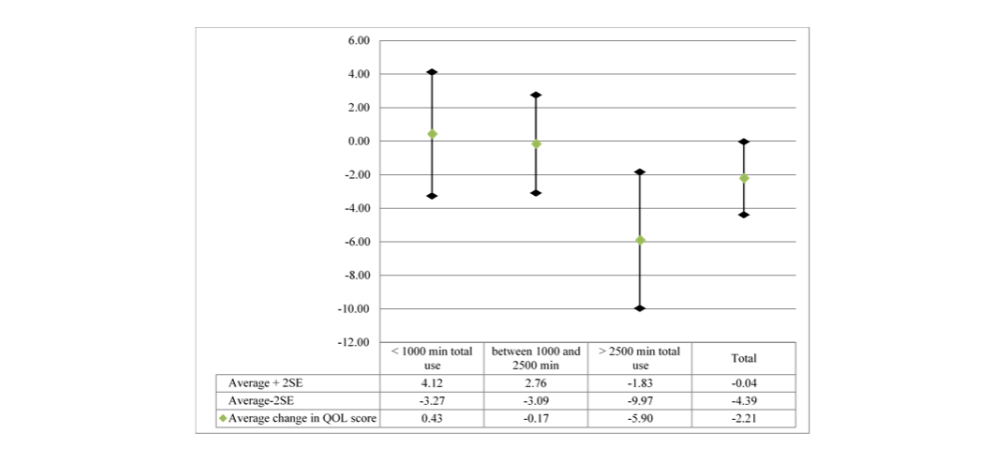
Changes in Quality of Life by Total Minutes using Music & Memory (iPod and iPad).

The QOL analysis was limited to 26 matched resident pairs with both a pre- and post-QOL score. Table 2 shows no significant differences across the ALCs in the pre- or post-QOL scores. At baseline, the average QOL score per ALC ranged from 19.2 (ALC 1) to 27.0 (ALC 4) indicating more positive resident QOL. Over a 3-month period, the change in residents’ QOL was significant (*P*=.047), and the differences across ALCs were also significant (*F*_25_=3.76, *P*=.02). Changes in QOL for individual residents are shown in Multimedia Appendix 7. Figure 2 shows the changes in QOL by total PMATE utilization over the 3-month period (April to June 2016). The difference between the groups is significant (*F*_25_=5.09, *P*=.02), with the differences between the high utilizers as compared with the low (*P*=.032) and medium (*P*=.035) utilizers of the PMATE program being significant. Results from the sensitivity analysis (not shown) confirmed these findings.

Residents’ use of medications by classification included antipsychotic (n=12), antidepressant (n=18), antianxiety (n=10), Alzheimer or dementia (n=9), or mood stabilizer (n=7). A total of 11 residents were on pro re nata (PRN) behavioral medications, and 2 residents went from scheduled to PRN medications. Although PRN medication use appeared to have decreased, the sample size was too small to draw conclusive evidence. However, the staff in 1 ALC was able to describe the impact of the PMATE program on 1 resident’s medication use, stating the following:

We had a lady here that was declining, pretty steadily cognitively, with her speech; she was just not talking much anymore. Her gait was declining. She was part of our program. She has become very alert and interactive again, talking again, more mobile again. I have been able to get her off all her behavioral meds. Her family just could not believe the huge turnaround that she had

**Table 1:** Total self-reported iPod and iPad use and minutes by residents within an assisted living community (ALC).

ALC and resident		iPod			iPad		
		Times utilized	Minutes utilized	Average minutes per use	Times utilized	Minutes utilized	Average minutes per use
**ALC 1 (total)**		**270^a^**	**7481**	**27.71**	**190**	**4462**	**23.48**
	1	63	1732	27.49	43	870	20.23
	2	52	1370	26.35	48	1610	33.54
	3	27	249	9.22	48	1302	27.13
	4	64	2000	31.25	33	465	14.09
	5	64	2130	33.28	18	215	11.94
**ALC 2 (total)**		**45**	**3280**	**72.89**	**55**	**1287**	**23.40**
	1	8	185	23.13	10	104	10.40
	2	1	30	30.00	13	128	9.85
	3	6	175	29.17	11	285	25.91
	4	7	95	13.57	6	150	25.00
	5^b^	5	2070	414.00	1	10	10.00
	6	7	395	56.43	6	415	69.17
	7	10	300	30.00	8	195	24.38
	8^b^	1	30	30.00	N/A^c^	N/A	N/A
**ALC 3 (total)**		**133**	**10,812**	**81.29**	**47**	**735**	**15.64**
	1	26	2272	87.38	8	170	21.25
	2	19	1505	79.21	8	135	16.88
	3	14	1740	124.29	N/A	N/A	N/A
	4	8	870	108.75	3	45	15.00
	5^b^	6	710	118.33	2	20	10.00
	6	14	750	53.57	11	170	15.45
	7	19	1225	64.47	8	115	14.38
	8	14	945	67.50	6	75	12.50
	9	13	795	61.15	1	5	5.00
**ALC 4 (total)**		**203**	**17,858**	**87.97**	**70**	**2405**	**34.36**
	1	33	2390	72.42	18	645	35.83
	2^b^	52	4388	84.38	11	375	34.09
	3	32	2910	90.94	15	520	34.67
	4	27	2155	79.81	14	495	35.36
	5^b^	57	5990	105.09	12	370	30.83
	6^b^	2	25	12.50	N/A	N/A	N/A
**ALC 5 (total)**		**233**	**24255**	**104.10**	**44**	**1035**	**23.52**
	1	41	5230	127.56	6	135	22.50
	2	42	5195	123.69	8	210	26.25
	3	26	2245	86.35	6	105	17.50
	4	32	2505	78.28	6	135	22.50
	5	29	2560	88.28	5	90	18.00
	6	37	4345	117.43	7	210	30.00
	7	26	2175	83.65	6	150	25.00
Grand total		**884**	**63,686**	**72.04**	**406**	**9924**	**24.44**

^a^Bolded values signify the total utilization (times and minutes) as well as the average time across all residents in the ALC.

^b^Data from 6 residents who passed away, were discharged, or refused to participate were excluded from subsequent analysis.

^c^N/A: not applicable.

**Table 2:** Residents’ quality of life (QOL)—pre, post, and changes over time by assisted living communities.

ALC^a^	Number of residents	Pre-QOL^b^, mean (95% CI)	Post-QOL^c^, mean (95% CI)	Change in QOL^d^, mean (95% CI)
ALC 1	5	19.20 (14.36 to 24.04)	16.00 (9.26 to 22.74)	−3.20 (−6.41 to 0.01)
ALC 2	6	21.00 (14.23 to 27.77)	21.67 (15.85 to 27.49)	0.67 (−3.87 to 5.20)
ALC 3	5	22.80 (17.73 to 27.87)	21.90 (17.73 to 26.07)	−0.90 (−2.57 to 0.77)
ALC 4	3	27.00 (6.67 to 47.33)	16.00 (7.04 to 24.96)	−11.00 (−23.91 to 1.91)
ALC 5	7	26.43 (20.99 to 31.87)	25.29 (16.31 to 34.26)	−1.14 (−7.03 to 4.72)
Total^e^	26	23.15 (20.84 to 25.46)	20.94 (18.23 to 23.65)	−2.21 (−4.28 to −0.14)

^a^ALC: assisted living community.

^b^No significant differences in pre-QOL across ALC (*P*=.18).

^c^No significant differences in post-QOL across ALC (*P*=.11).

^d^Change in QOL differs significantly by ALC (*P*=.02).

^e^Overall residents’ QOL decreased significantly by 2.21 points (*P*=.047).

## Discussion

### Principal Findings

The study is one of the first to explore the impact of the Music & Memory PMATE program on residents living in ALCs. Findings suggest that higher utilization over time improves residents’ QOL and that use of the PMATE program might be related to changes in agitation. The QOL for individuals with dementia in ALCs is similar to other individuals living with dementia who reside in NHs or other long-term care settings [58-62]. Our results found significant positive changes in QOL were influenced by PMATE exposure. Earlier studies suggested no or worsening change in QOL for individuals with dementia post pharmaceutical and nonpharmaceutical intervention implementation [61-65]. Application of the comfort versus discomfort subscales in our sample [66] showed a greater reduction, on average, of discomfort (−1.96) versus comfort (−0.36) in residents’ QOL. Residents with the highest PMATE exposure (>2500 min) showed similar results.

Elements of discomfort observed in the study included emotional expressions such as appears sad, cries, has facial expression of discomfort, appears physically uncomfortable, verbalizes discomfort, irritable, or aggressive. Residents displaying these negative emotions exhibited improvements after utilization of the PMATE program, more so than residents who were already enjoying elements of comfort. Elements of comfort include the following: appears calm and comfortable, smiles, enjoys eating, enjoys touching or being touched, and enjoys interacting with others. In this subscale, the technological and nonpharmacologic PMATE intervention contributes to positive mood management through person-centered care for residents. Our study results provide promising evidence that PMATE exposure positively impacts residents’ QOL; however, further research is needed in a larger sample of residents to confirm these findings.

In our study population, PMATE exposure had no significant impact on changes in residents’ agitation. Although residents’ agitation across the sample decreased over time, these changes appear to be a resident-specific phenomenon. The true impact of the PMATE program on residents’ agitation levels may be masked by the small sample size and the inadequate reporting of the intervention used. For individual residents, PMATE use may result in a decrease in antipsychotic medication dependence. Despite these findings, staff reported that residents’ PMATE participation did impact the residents as well as their interactions with them. As the staff in one ALC stated:

You could just see immediately, the resident would light up and it would even calm the staff somewhat because if the resident is very tense, and you do everything you can think of to help this resident, and then you put the music on, and if it relaxes them, I think it in turn helps the staff

Another ALC expressed the positive impact on staff saying that “it gets them more involved with the residents and getting to know how they interact, what changes mood.” However, a more comprehensive study across multiple ALCs is needed to confirm these preliminary findings.

### Limitations

The study has several limitations. The small resident sample size (n=26) and a missing workbook from one ALC, which could have provided data for 6 additional residents, are a couple of the limitations. Self-reporting of resident outcomes and PMATE utilization, especially iPod use, represented another study limitation. Future studies should explore automated data collection for iPod and iPad utilization, which would decrease staff burden and increase accuracy. Missing data, especially for medication use, was another limitation. Depending on the sample size, chart reviews of medication use, or electronic health record extractions should increase the accuracy of changes in medication use over time for ALC residents. Finally, the study was limited to a 3-month period, which was not an adequate time period to assess antipsychotic medication use and implement changes based on the residents’ PMATE interactions.

### Conclusions

The use of nonpharmacological interventions, such as personalized music, is intended to maintain cognitive function, improve QOL, and help mitigate individual behavioral issues for older adults with ADRD. This pilot study represents a foundational step, providing researchers and practitioners with evidence that offering access to Music & Memory’s PMATE program may be effective in improving outcomes associated with behavioral and psychological symptoms of dementia for residents living with ADRD in ALCs. These benefits, by virtue of their interactions with residents, are transferred to ALC staff through an enhanced sense of trust and improved relationships. Future research across a larger sample of ALC residents is needed to evaluate the impact of intervention dose levels on residents’ outcomes and to identify effective approaches to implementing and sustaining the PMATE program in ALCs.

## Appendix

Multimedia Appendix 1 Assisted living community application.

Multimedia Appendix 2 Attributes of participating assisted living communities.

Multimedia Appendix 3 Tablet engagement apps.

Multimedia Appendix 4 Park Family Foundation project timeline.

Multimedia Appendix 5 Project evaluation binder.

Multimedia Appendix 6 iPad app utilization and average usefulness of the app in promoting resident engagement.

Multimedia Appendix 7 Changes in resident level quality of life.

## Abbreviations

ADRD: Alzheimer disease or related dementia
ALC: assisted living community
ANOVA: analysis of variance
BPSD: behavioral and psychological symptoms of dementia
DHS: Department of Health Services
NH: nursing home
PAS: Pittsburgh Agitation Scale
PMATE: personalized music and tablet engagement
PRN: pro re nata
QOL: quality of life
QUALID: Quality of Life in Late Stage Dementia
WCCEAL: Wisconsin Coalition for Collaborative Excellence in Assisted Living
