# Supplementary material for: Evaluating the Impact of Music & Memory’s Personalized Music and Tablet Engagement Program in Wisconsin Assisted Living Communities: Pilot Study
Source: JMIR Aging. 2019 Mar 14;2(1):e11599. doi: 10.2196/11599 (PMC6716484; doi:10.2196/11599)
Supplement: Multimedia Appendix 1 [file aging_v2i1e11599_app1.pdf]

ASSISTED LIVING APPLICATION

Assisted Living Application

This application is to be completed by Wisconsin Coalition for Collaborative Excellence (WCCEAL) members, in good standing, who have been referred by their association to participate in the **Wisconsin Music & Memory + iPad Program**. The project is designed to fully fund the certification of the assisted living community as a Music & Memory facility with additional training on implementing an iPad in their community. In addition, the community will be provided equipment for an average of 6 residents/tenants from each community to participate in the program. The training, certification, and all equipment will be provided by MUSIC & MEMORY^{SM} at no cost to the facility --- a value of over $1,500 to each community. One of the goals of this project is to reduce the reliance on anti-psychotic and anti-anxiety medications for people with Dementia. *This program is generously supported by the Neil E. Park Family Foundation.*

Orientation

There will be an orientation webinar available to learn more about this opportunity. The video conference will be held December 17, 2015 from 1:00-2:00 p.m. CST via Adobe Connect Pro Link:
https://connect.wisconsin.gov/dhs-music-memory/
All you need to do is click the link a few minutes prior to the event and log in as a "guest" and enter your community or agency name. You will be able to chat with others, send questions and participate in poll questions, etc. **Make sure the sound on your computer is on**. It is recommended that you test your computer prior to attending a meeting. You can do this by going to https://connect.wisconsin.gov/common/help/en/support/meeting_test.htm .

Training

Communities are required to designate one (1) staff person to serve as the Lead Project Person. This person will serve as liaison between the Wisconsin Music & Memory Program, the Center for Health Systems Research & Analysis (CHSRA) and the assisted living community. The Lead Project Person will be expected to attend a training which consists of three 90-minute sessions. These training sessions will be provided via webcast by the MUSIC & MEMORY^{SM} founding Executive Director, Dan Cohen, MSW, who will teach staff how to implement the program within their facility. This three-session training will be offered during the following times:

- **February 16, 17 and 18: 12:00-1:30 p.m. CST or**
- **March 15, 16 and 17: 12:00-1:30 p.m. CST**

In addition, staff will receive training on the operation and implementation of the iPad and the associated apps. This training will be offered **Wednesday March 2, 2016 from 1:30-3:00 pm**. If the Lead Project Person is unable to attend a training session, the community may designate an alternate staff person to attend. Communities are also encouraged to have all interested staff attend, as greater success has been seen in facilities when multiple staff members receive training. Communities will be awarded certification as a Music & Memory facility upon completion of the staff training. To learn more, visit the Wisconsin Music & Memory webpage at https://musicandmemory.org/landing/music-memory-certification-program1/.

Equipment

After completion of the training for both the Music & Memory and the iPads, MUSIC & MEMORY^{SM} will send (via U.S. Mail) to each certified Music & Memory assisted living community the equipment needed to implement the program. Each facility will receive a $100 iTunes gift card, one set of external speakers, one headphone splitter, and an average of 6 iPod Shuffles, headphones, and AC adapters for resident/tenant use. In addition, each community will receive an iPad with appropriate applications.

Evaluation

An evaluation conducted by the Center for Health Systems Research & Analysis (CHSRA) will assess the impact of the program on the residents and the assisted living community. Assisted living communities will agree to participate in an evaluation by completing periodic questionnaires regarding the effect the Music & Memory program has on residents and staff and other requirements by the research agency.

Ongoing Support

The Department of Health Services will host monthly support meetings using video conferencing open to all participating communities to discuss project successes and to work through challenging issues.

Instructions

Complete all portions of the application.
Submit your request electronically --- no later than **December 31, 2015** to wcceal@chsra.wisc.edu

- If you have questions regarding the Wisconsin Music & Memory Program or the application process, please contact DHS staff, Kevin Coughlin at (608) 266-6989 or e-mail us at DHSMusicMemory@dhs.wisconsin.gov.

WISCONSIN MUSIC AND MEMORY PROGRAM + iPad
ASSISTED LIVING COMMUNITY APPLICATION

Assisted Living Community Name			Licenses Beds	License Number
Address	City	State	Zip Code	
Name – **Administrator/Manager**	Email Address	Telephone Number		

Name – **Lead Project Person**	Title
Email Address	Telephone Number

Name – **Alternate Project Person**	Title
Email Address	Telephone Number

Development of the individualized playlist for each resident is greatly enhanced by using a laptop computer with the resident. Does your community have a laptop computer?	☐ Yes ☐ No

Each facility will be sent equipment for an average of 6 residents/tenants to participate in the program.

How many residents/tenants in your community with a diagnosis of dementia or Alzheimer's Disease?	
How many residents/tenants with a diagnosis of dementia or Alzheimer's Disease are also receiving anti-psychotic or anti-anxiety medications?	
Provide the number of residents you anticipate will participate in the program.	

Terms of Participation

- At a minimum, one staff person must attend each of the three Music & Memory training sessions. Multiple staff members are encouraged to attend the trainings.
- Participate in an evaluation of the project as requested by the research agency. (We will work with the research agency to keep the information requested to a reasonable amount)
- Implement the Music & Memory program as outlined in the training.

☐ Yes ***As Administrator of the above-named assisted living community, I agree to the Terms of Participation listed above.***

Administrator	Date
