# Supplementary material for: Evaluating the Impact of Music & Memory’s Personalized Music and Tablet Engagement Program in Wisconsin Assisted Living Communities: Pilot Study
Source: JMIR Aging. 2019 Mar 14;2(1):e11599. doi: 10.2196/11599 (PMC6716484; doi:10.2196/11599)
Supplement: Multimedia Appendix 2 [file aging_v2i1e11599_app2.pdf]

Appendix 2: Attributes of Participating Assisted Living Communities

Community	Type	# Licensed Beds	City	# of Residents with Alzheimer/Dementia	# of Residents with Alzheimer/Dementia on Medications	Estimated Number of Study Participants
Heritage Gardens	CBRF	18	Green Bay	18	6	14
Pine Villa	CBRF	14	Prairie Du Sac	14	9	9
Remembrance Home	CBRF	19	Beaver Dam	19	7	8
Germantown Matterhaus	RCAC	26	Germantown	26	8	8
Sylvan Crossings Fitchburg	CBRF	19	Fitchburg	19	9	3/week/rotating
The Cottages MC	CBRF	14	Shawano	14	9	6

CBRF: Community Based Rehabilitation Facility

RCAC: Residential Care Apartment Complex
