# Supplementary material for: Evaluating the Impact of Music & Memory’s Personalized Music and Tablet Engagement Program in Wisconsin Assisted Living Communities: Pilot Study
Source: JMIR Aging. 2019 Mar 14;2(1):e11599. doi: 10.2196/11599 (PMC6716484; doi:10.2196/11599)
Supplement: Multimedia Appendix 3 [file aging_v2i1e11599_app3.pdf]

App	Vendor	Category	Description	Impact	Adult or Child oriented
**Osmo Words**	PlayOsmo	Games	Mirror projects game to table. Word association game with physical component.	Sense of accomplishment	Child
**Osmo Tangram**	PlayOsmo	Games	Mirror projects game to table. Physical puzzle game.	Sense of accomplishment	Child
**Puzzable**	Brainwave Games	Games	Customizable jigsaw puzzle.	Sense of accomplishment	Youth/Adult
**Google Images**	Google	Images	Internet image searches.	Reminiscing / Socialization	Adult
**Google Earth**	Google Earth	Images	Worldwide location searches.	Reminiscing / Socialization	Adult
**Adobe Spark**	Adobe	Images	Create narrated visual stories.	Reminiscing / Socialization	Adult
**Ted Talks**	TED	Lifelong Learning	Short talks on topics of interest.	Sense of accomplishment	Adult
**Art in the Moment**	CJE Senior Life	Lifelong Learning	Questions for conversations about art.	Sense of accomplishment	Adult

App	Vendor	Category	Description	Impact	Adult or Child oriented
**Pocket Pond**	TriggerWave	Relaxation	Koi pond water and fish react to touch with immediate feedback.	Relaxation	Adult
**SingFit**	Music Health Technologies	Relaxation	Guide singer and lyric coach cue words for singalong.	Relaxation / Socialization	Adult
**Garage Band**	Apple Inc.	Relaxation	Tool for creating music.	Relaxation / sense of accomplishment	Both
**Colorfy**	TFG Co.	Relaxation	Adult coloring app allows user to choose image, color and autofill picture.	Relaxation / sense of accomplishment	Adult
**Pottery Lite**	Infinite Dreams	Relaxation	Simulates creating ceramics on wheel.	Relaxation / sense of accomplishment	Adult
**NatureSpace**	Holographic Audio Theater	Relaxation	Sounds of Nature	Relaxation	Adult
**Take A Break**	Meditation Oasis	Relaxation	Guided Meditation	Relaxation	Adult
