# Supplementary material for: Evaluating the Impact of Music & Memory’s Personalized Music and Tablet Engagement Program in Wisconsin Assisted Living Communities: Pilot Study
Source: JMIR Aging. 2019 Mar 14;2(1):e11599. doi: 10.2196/11599 (PMC6716484; doi:10.2196/11599)
Supplement: Multimedia Appendix 4 [file aging_v2i1e11599_app4.pdf]

	Task	Start	End	Dur	2015	2016			
					Q4	Q1	Q2	Q3	Q4
	Wisconsin Assisted Living Park Family Foundation	12/15/15	7/9/16	201					
1	Project Orientation Webinar	12/15/15	12/15/15	1					
2	Music and Memory IPod Training (Option 1)	2/16/16	2/18/16	3					
3	Music and Memory IPad training	3/2/16	3/2/16	1					
4	Music and Memory IPod Training (Option 2)	3/15/16	3/17/16	3					
5	Project Evaluation Webinar	3/29/16	3/29/16	1					
6	Music and Memory Implementation Period	4/1/16	6/30/16	90					
7	Initial Quality of Life Data Collection Period	4/4/16	4/8/16	5					
8	Initial Pittsburgh Agitation Scale Data Collection Period	4/4/16	4/8/16	5					
9	2nd Pittsburgh Agitation Scale Data Collection Period	5/2/16	5/6/16	5					
10	3rd Pittsburgh Agitation Scale Data Collection Period	6/6/16	6/10/16	5					
11	4th Pittsburgh Agitation Scale Data Collection Period	7/4/16	7/9/16	5					
12	Final Quality of Life Data Collection Period	7/4/16	7/9/16	5					
13	Data Collection for IPod and IPad Use	4/1/16	6/30/16	90					
14	Data Collection for resident anti-psychotic medication use	3/1/16	6/30/16	120					
