# Supplementary material for: Evaluating the Impact of Music & Memory’s Personalized Music and Tablet Engagement Program in Wisconsin Assisted Living Communities: Pilot Study
Source: JMIR Aging. 2019 Mar 14;2(1):e11599. doi: 10.2196/11599 (PMC6716484; doi:10.2196/11599)
Supplement: Multimedia Appendix 5 [file aging_v2i1e11599_app5.pdf]

Park Family Foundation
Music & Memory Evaluation
April - July 2016

Jay's House - Madison

Evaluation conducted by:

Center for Health Systems Research & Analysis (CHSRA)
University of Wisconsin - Madison

Helpdesk email: wcceal@chsra.wisc.edu
Phone: 1-888-300-8098

Please assign each resident participant a unique identification #. This # will correspond with the ID numbers on the evaluations in the resident section of your packet. Complete the forms with the corresponding ID # assigned to that resident. REMOVE THIS PAGE before returning your binder to CHSRA.

ALC Name	Resident Name	Resident ID
Jay's House Madison		9-201
Jay's House Madison		9-202
Jay's House Madison		9-203
Jay's House Madison		9-204
Jay's House Madison		9-205
Jay's House Madison		9-206
Jay's House Madison		9-207
Jay's House Madison		9-208

Park Family Foundation Music & Memory Evaluation

April-July 2016

Resident Name/ID Crosswalk

(Front pocket – for your reference, do not return with binder)

Community Section-Informational

Pittsburgh Agitation Scale Instructions
Qualid Instructions
Psychotropic Medication List

Resident Section-To Complete

IPOD Tracking Log (1 per resident)
IPAD Usage Log (1 per resident)
Medication Tracking Log (1 per resident)
On-going

Pittsburgh Agitation Scale

(4 per resident)
Observation Period 1: April 4-8, 2016
Observation Period 2: May 2-6, 2016
Observation Period 3: June 6-10, 2016
Observation Period 4: July 4-8, 2016

Quality of Life in Late Stage Dementia

(2 per resident)
Observation Period 1: April 4-8, 2016
Observation Period 4: July 4-8, 2016

CHSRA HelpDesk:
wcceal@chsra.wisc.edu
1-888-300-8098

Postage Paid Return Envelope for return of
[illegible]

iPod Tracking Log

<u>Instructions</u>:
1. Please use this sheet to track the resident's use of the iPod.
2. We are asking that you record each time that the iPod is checked out and returned for this resident.
3. Please complete the following information each time that the iPod is used **throughout the duration of the study.**

iPad Usage Log

<u>Instructions</u>:
For any app utilized with each resident during an iPad session, please rank the usefulness of the app at promoting resident engagement using the following scale:
- Very Useful – 4
- Somewhat Useful – 3
- Not Very Useful – 2
- Not at All Useful -1

Please complete this information eact time the iPad is used **throughout the duration of the study.**

Anti-psychotic Medication Tracking Log

<u>Instructions</u>:
1. Please use this sheet to track the use of **anti-psychotic medications** for each resident.
2. Please record each medication for the resident on a separate line.
3. Initial entries should be any anti-psychotic medications that the resident was prescribed and using on or before **April 1, 2016.**
4. Please track medication use **throughout the duration of the study.**

Pittsburgh Agitation Scale (PAS) Instructions

The Pittsburgh Agitation Scale allows an observer to rate the severity of agitation related to dementia according to four general behavior groups:

1. Aberrant Vocalizations,
2. Motor Agitation,
3. Aggressiveness and
4. Resisting Care across four dimensions: Washing, Dressing, Eating and Taking Medications.

Each of the behavioral groups are anchored on a scale from 0 to 4 where 0 represent the lower end of the scale and 4 is the higher end of the scale.

The scores of Aberrant Vocalization and Motor Agitation are assigned based on intensity, disruptiveness within the environment, and the ease with which the patient's behavior can be redirected.

Scores on the Aggression and Resistance to Care scales are only taken into account within those particular behavior groups.

Instructions: The observer will conduct a weekly assessment of the resident to determine and rate their behaviors utilizing the behavior anchors (examples) to assign the highest possible score (most severe) seen within the defined rating time (which can range from 1 to 8 hours).

For this project, we are asking that you conduct a weekly assessment (Monday through Friday) of each resident to determine and rate their behaviors.

The process will be completed by observing each resident over a defined rating time which can range from 1 to 8 hours.

At the end of each daily observation period, the staff should rate and record the residents' level of agitation for that day for each behavioral groups that best describes the residents' behavior.

The rating should be completed utilizing the specific behavior anchors to assign the highest possible score (most severe) observed for the resident within the defined rating time.

*Please note the specific instructions for the Aggression Behavioral Group and if the behavior is only present when resisting care, the residents score in the Aggression Behavioral Group should be marked as "**Not Present**"*

In addition to the residents' behavior, the observer will record the hours of sleep during the rating period, as well as they will check if use of physical restraints, PRN medications, Music & Memory or other interventions were specifically utilized in an effort to control a resident's agitation.

The observation process will be repeated monthly during the first full week of each month from April through July 2016.

If possible, the observations and ratings should be completed by the same individual.

The observation dates are:

- Period 1: April 4-8, 2016
- Period 2: May 2-6, 2016
- Period 3: June 6-10, 2016
- Period 4: July 4-8, 2016

Quality of Life in Late Stage Dementia (QUALID) Instructions

The QUALID should be completed by professional caregiver who has regular contact and is most familiar with the resident's general behavior. The individual completing the QUALID must have spent a significant portion of at least 3 days out of the last 7 days with the resident, in order to accurately rate the items on the scale.

You will be asked to rate an individual's behaviors across eleven difference dimensions utilizing the responses within each question.

Please note that there is no one right or wrong answer, it is more important to know how you would rate his/her behavior from your observations.

Specifically, we want to know about his/her behavior over the past week only, not how he/she previously behaved. Please remember that your answers should reflect his/her behavior over the past seven days.

If you have difficulty choosing a rating for an item, just make your best guess. Again, indicate your observation about his/her behavior over the past week.

For this study, we are asking that you complete the QUALID for each resident at two points in time.

- **The initial rating should be completed on either April 11 or 12 and should reflect the resident behavior during the week of April 3 to April 8, 2016- Period 1.**
- **The final rating period should be completed on either July 12 or 13 and should reflect the resident behavior during the week July 4- 8, 2016- Period 4.**

Psychotropic Medications

Chapter 55.01 Definitions. "Psychotropic medication" means a prescription drug, as defined in s.450.01 (20), that is used to treat or manage a psychiatric symptom or challenging behavior.

Some psychotropic medications fall into specific medication classes like antipsychotics or antidepressants. In other cases, the medications may be primarily used for other diseases but have been found effective in controlling behaviors thus making that specific use a psychotropic medication.

Below is a list of psychotropic medications or medications with psychotropic uses. This list is not intended to be all-inclusive, however it can act as a resource to alert you to psychotropic medications where regulations like psychotropic monitoring, informed consent or guardianship may impact. If you have any questions about the medication, its intended use or adverse effects please contact the prescribing physician, pharmacist or nurse.

* Sorted alphabetically by Brand Name

Brand Name	***Generic Name***	***Class***
Abilify	Aripiprazole	Antipsychotics
Clozaril	Clozapine	Antipsychotics
Compazine	Prochlorperazine	Antipsychotics
Fanapt	Iloperidone	Antipsychotics
FazaClo	Clozapine	Antipsychotics
Geodon	Ziprasidone	Antipsychotics
Haldol	Haloperidol	Antipsychotics
Invega	Paliperidone	Antipsychotics
Latuda	Lurasidone	Antipsychotics
Loxitane	Loxapine	Antipsychotics
Mellaril	Thioridazine	Antipsychotics
Moban	Molindone	Antipsychotics
Navane	Thiothixene	Antipsychotics
Orap	Pimozine	Antipsychotics
Prolixin	Fluphenazine	Antipsychotics
Risperdal	Risperidone	Antipsychotics
Saphris	Asenapine	Antipsychotics
Serentil	Mesoridazine	Antipsychotics
Seroquel	Quetiapine	Antipsychotics
Sparine	Promazine	Antipsychotics
Stelazine	Trifluoperazine	Antipsychotics
Symbyax	Fluoxetine & Olanzapine	Antipsychotics
Taractin	Chlorprothixene	Antipsychotics
Thorazine	Chlorpromazine	Antipsychotics
Tindal	Acetophenazine	Antipsychotics
Trilafon	Perphenazine	Antipsychotics
Vesprin	Triflupromazine	Antipsychotics
Zyprexa	Olanzapine	Antipsychotics

iPod Tracking Log for:

Instructions:

1. Please use this sheet to track this resident's use of the iPod.
2. We are asking that you record each time that the iPod is checked out and returned for this resident.
3. Please complete the following information each time that the iPod is used from April 4, 2016 through June 30, 2016

	Date (mm/dd/yy)	Time Signed Out	Time Returned	Duration of iPod Use (in Minutes)
Example	3/30/2016	10:15 AM	11:00 AM	45

FOR:

Instructions: **For any app utilized with the resident during this iPad session, please rank the usefulness of the app at promoting resident engagement utilizing the following scale:**

Very Useful - 4
Somewhat Useful - 3
Not Very Useful - 2
Not at All Useful -1

			**Games**		**Life Stories**			**Relaxation**						**Lifelong Learning**	
	Date	Duration (in Minutes)	Words-Osmo	Tangram-Osmo	Swaha	Google Earth	Image Search	Pocket Pond	Sing Fit	Colorfy	Pottery Lite	Naturespace	Take a Break	Ted Talks	Art in the Moment
Example	3/30/2016	45	3			2			1						

Anti-Psychotic Medication Tracking Log for:

Instructions:
1. Please use this sheet to track the use of **anti-psychotic medications** for this resident.
2. Please record each medication for the resident on a separate line.
3. Initial entries should be any anti-psychotic medications that the resident was prescribed and using on or before April 1, 2016.
4. Please track medication use from April 4, 2016 through June 30, 2016

	Anti-psychotic Medication	Start Date (mm/dd/yy)	Dose	Frequency	Discontinuance Date (mm/dd/yy)
Example	Haldol	3/31/2016	3 mg	4 to 8 hr PRN	6/24/2016
Example	Moban	4/24/2016	25 mg	3 x per day	6/30/2016

**Observation Period 4** Pittsburgh Agitation Scale for:					

Instructions:

1. Please check only the highest intensity score for each behavior group that you observed during each day of the rating period. Use the anchor points as a guide to choose a suitable level of severity. Anchors on the left side of the scale (0) are less intense actions than those on the right side of the scale (4).
2. Not all anchor points need be present. Choose the more severe level when in doubt.
3. Only one column or choice should be made for each day during the week long observation period.

**1. Please provide the following information about the resident for each day of the observation period**	How many hours of sleep did resident get?	When did the observation period start?	When did the observation period end?
July 4 2016			
July 5 2016			
July 6 2016			
July 7 2016			
July 8 2016			

	Intensity During Rating Period				
**2. Aberrant Vocalization** (repetitive requests or complaints, nonverbal. vocalizations, e.g., moaning, screaming)	0. Not present	1. Low volume, not disruptive in milieu, including crying	2. Louder than conversational, mildly disruptive, redirectable	3. Loud, disruptive, difficult to redirect	4. Extremely loud screaming or yelling, highly disruptive, unable to redirect
July 4 2016	☐	☐	☐	☐	☐
July 5 2016	☐	☐	☐	☐	☐
July 6 2016	☐	☐	☐	☐	☐
July 7 2016	☐	☐	☐	☐	☐
July 8 2016	☐	☐	☐	☐	☐

	Intensity During Rating Period				
**3. Motor Agitation** (pacing, wandering, moving in chair, picking at objects, disrobing, banging on chair, taking others' possessions. Rate "intrusiveness" by normal social standards, not by effect on other patients in milieu. If "intrusive" or "disruptive" due to noise, rate under "Vocalization.")	0. Not present	1. Pacing or moving about in chair at normal rate (appears to be seeking comfort, looking for spouse, purposeless movements)	2. Increased rate of movements, mildly intrusive, easily redirectable	3. Rapid movements, moderately intrusive or disruptive, difficult to redirect	4. Intense movements extremely intrusive or disruptive, not redirectable verbally
July 4 2016	☐	☐	☐	☐	☐
July 5 2016	☐	☐	☐	☐	☐
July 6 2016	☐	☐	☐	☐	☐
July 7 2016	☐	☐	☐	☐	☐
July 8 2016	☐	☐	☐	☐	☐

	Intensity During Rating Period				
**4. Aggressiveness (<u>mark "not present" if aggressive only when resisting care</u>)**	0. Not present	1. Verbal Threats	2. Threatening gestures, no attempt to strike	3. Physical towards property	4. Physical towards self or others
July 4 2016	☐	☐	☐	☐	☐
July 5 2016	☐	☐	☐	☐	☐
July 6 2016	☐	☐	☐	☐	☐
July 7 2016	☐	☐	☐	☐	☐
July 8 2016	☐	☐	☐	☐	☐
	Intensity During Rating Period				
**5. Resisting Care related to Washing**	0. Not present	1. Procrastination or avoidance	2. Verbal/gesture of refusal	3. Pushing away to avoid task	4. Striking out at caregiver
July 4 2016	☐	☐	☐	☐	☐
July 5 2016	☐	☐	☐	☐	☐
July 6 2016	☐	☐	☐	☐	☐
July 7 2016	☐	☐	☐	☐	☐
July 8 2016	☐	☐	☐	☐	☐
	Intensity During Rating Period				
**6. Resisting Care related to Dressing**	0. Not present	1. Procrastination or avoidance	2. Verbal/gesture of refusal	3. Pushing away to avoid task	4. Striking out at caregiver
July 4 2016	☐	☐	☐	☐	☐
July 5 2016	☐	☐	☐	☐	☐
July 6 2016	☐	☐	☐	☐	☐
July 7 2016	☐	☐	☐	☐	☐
July 8 2016	☐	☐	☐	☐	☐
	Intensity During Rating Period				
**7. Resisting Care related to Eating**	0. Not present	1. Procrastination or avoidance	2. Verbal/gesture of refusal	3. Pushing away to avoid task	4. Striking out at caregiver
July 4 2016	☐	☐	☐	☐	☐
July 5 2016	☐	☐	☐	☐	☐
July 6 2016	☐	☐	☐	☐	☐
July 7 2016	☐	☐	☐	☐	☐
July 8 2016	☐	☐	☐	☐	☐

	Intensity During Rating Period				
**8. Resisting Care related to Taking of Medications**	0. Not present	1. Procrastination or avoidance	2. Verbal/gesture of refusal	3. Pushing away to avoid task	4. Striking out at caregiver
July 4 2016	☐	☐	☐	☐	☐
July 5 2016	☐	☐	☐	☐	☐
July 6 2016	☐	☐	☐	☐	☐
July 7 2016	☐	☐	☐	☐	☐
July 8 2016	☐	☐	☐	☐	☐
**9. Were any of the following used during this rating period because of behavior problems? (<u>Please check all of the interventions that apply)</u>**	Seclusion	PRN Meds	Restraints	Music and Memory Interventions	Other Interventions (Please specify below)
July 4 2016	☐	☐	☐	☐	☐
July 5 2016	☐	☐	☐	☐	☐
July 6 2016	☐	☐	☐	☐	☐
July 7 2016	☐	☐	☐	☐	☐
July 8 2016	☐	☐	☐	☐	☐

Instructions: For each question, please rate the residents behaviors using the responses provided. There is no one right or wrong answer, it is important to know how you would rate his/her behavior from your observations. Specifically, we want to know about his/her behavior over the past week only, not how he/she previously behaved. Which response best describes ________ over the past week**:**

A. **Resident smiles**

- ☐ 1. Spontaneously once or more each day
- ☐ 2. Spontaneously less than once each day
- ☐ 3. Only in response to external stimuli; at least once each day
- ☐ 4. Only in response to external stimuli; less than once each day
- ☐ 5. Rarely, if at all

B. **Resident appears sad**

- ☐ 1. Rarely or never
- ☐ 2. Only in response to external stimuli; less than once each day
- ☐ 3. Only in response to external stimuli; at least once each day
- ☐ 4. for no apparent reason less than once each day
- ☐ 5. for no apparent reason once or more each day

C. **Resident cries**

- ☐ 1. Rarely or never
- ☐ 2. Only in response to external stimuli; less than once each day
- ☐ 3. Only in response to external stimuli; at least once each day
- ☐ 4. for no apparent reason less than once each day
- ☐ 5. for no apparent reason once or more each day

OVER

D. **Resident has a facial expression of discomfort - appears unhappy or in pain (looks worried, grimaces, furrowed or turned down brow)**

☐ 1. Rarely or never

☐ 2. Less than once each day

☐ 3. At least once each day

☐ 4. Nearly half of each day

☐ 5. Most of each day

E. **Resident appears physically uncomfortable –he/she squirms, writhes, frequently changes position**

☐ 1. Rarely or never

☐ 2. Less than once each day

☐ 3. At least once each day

☐ 4. Nearly half of each day

☐ 5. Most of each day

F. **Resident makes statements or sounds that suggest discontent, unhappiness or discomfort (complains, groans, screams)**

☐ 1. Rarely or never

☐ 2. Only in response to external stimuli; less than once each

☐ 3. Only in response to external stimuli; at least once each day

☐ 4. Without cause less than once each day

☐ 5. without cause once or more each day

G. **Resident is irritable or aggressive (becomes angry, curses, pushes or attempts to hurt others)**

☐ 1. Rarely or never

☐ 2. Only in response to external stimuli; less than once each

☐ 3. Only in response to external stimuli; at least once each day

☐ 4. Without cause less than once each day

☐ 5. Without cause once or more each day

H. **Resident enjoys eating**

☐ 1. At most meals and snacks

☐ 2. Twice a day

☐ 3. At least once a day

☐ 4. Less than once each day

☐ 5. Rarely or never

I. **Resident enjoys touching/being touched**

☐ 1. Almost always; almost always initiates touching/interaction with others

☐ 2. More than half the time; sometimes initiates touching//interaction with others

☐ 3. Half the time; never initiates touching, but doesn't resist touching/interaction with others

☐ 4. Less than half the time; often or frequently resists touching/being touched/interaction with others

☐ 5. Rarely or never; almost always resists touching/being touched/interaction with others

OVER

J. **Resident enjoys interacting or being with others**

☐ 1. Almost always; almost always initiates touching/interaction with others

☐ 2. More than half the time; sometimes initiates touching//interaction with others

☐ 3. Half the time; never initiates touching, but doesn't resist touching/interaction with others

☐ 4. Less than half the time; often or frequently resists touching/being touched/interaction with others

☐ 5. Rarely or never; almost always resists touching/being touched/interaction with others

K. **Resident appears emotionally calm and comfortable**

☐ 1. Most of each day

☐ 2. More than half of each day

☐ 3. Half of each day

☐ 4. Less than half of each day

☐ 5. Rarely or never
