# Supplementary material for: Evaluating the Impact of Music & Memory’s Personalized Music and Tablet Engagement Program in Wisconsin Assisted Living Communities: Pilot Study
Source: JMIR Aging. 2019 Mar 14;2(1):e11599. doi: 10.2196/11599 (PMC6716484; doi:10.2196/11599)
Supplement: Multimedia Appendix 6 [file aging_v2i1e11599_app6.pdf]

iPad Utilization All Residents [a]															
	**Games**			Life **stories**			Lifelong **learning**		**Relaxation**						
	Words Osmo	Tangram Osmo	Puzzable	Adobe Voice	Google Earth	Image Search	Ted Talks	Art in the Moment	Pocket Pond	Sing Fit	Garage Band	Colorfy	Pottery Lite	Nature space	Take a Break
Total Times App Used	43	15	112	16	23	114	44	2	59	47	31	85	25	30	21
Average Times Resident Used App	3.58	1.36	6.59	2.00	1.71	4.75	6.29	1.00	2.36	2.61	2.58	5.67	1.79	2.14	1.75
# of Residents using App	12.00	11.00	17.00	8.00	14.00	24.00	7.00	2.00	25.00	18.00	12.00	15.00	14.00	14.00	12.00
% of Residents using App	36%	33%	52%	24%	42%	73%	21%	6%	76%	55%	36%	46%	42%	42%	36%
Resident App use (Minimum)	1.00	1.00	1.00	1.00	1.00	1.00	1.00	1.00	1.00	1.00	1.00	1.00	1.00	1.00	1.00
Resident App use (Maximum)	16.00	3.00	33.00	5.00	3.00	19.00	21.00	1.00	6.00	7.00	4.00	26.00	3.00	4.00	5.00

Average App Usefulness	1.89	1.45	2.22	2.38	1.76	2.72	2.41	1.00	2.27	2.28	2.69	2.07	2.20	2.22	2.46
Standard Error App Usefulness	0.37	0.21	0.24	0.42	0.26	0.20	0.51	0.00	0.18	0.24	0.34	0.22	0.34	0.29	0.35
**iPad Utilization Excluding Residents Discharged, Refused to Participate or Passed Away** [b]															
	**Games**			Life **stories**			Lifelong **learning**		**Relaxation**						
	Words Osmo	Tangram Osmo	Puzzable	Adobe Voice	Google Earth	Image Search	Ted Talks	Art in the Moment	Pocket Pond	Sing Fit	Garage Band	Colorfy	Pottery Lite	Nature space	Take a Break
Total Times App Used	43	12	111	14	24	101	44	2	58	46	31	84	22	29	18
Average Times Resident Used App	3.31	1.20	6.53	2.00	1.60	4.81	5.50	0.67	2.32	2.56	2.38	5.60	1.83	2.07	1.64
# of Residents using App	12.00	9.00	16.00	6.00	14.00	20.00	7.00	2.00	24.00	17.00	12.00	14.00	11.00	13.00	10.00
% of Residents using App	43%	32%	57%	21%	50%	71%	25%	7%	86%	61%	43%	50%	39%	46%	36%

Resident App use (Minimum)	1.00	1.00	1.00	1.00	1.00	1.00	1.00	1.00	1.00	1.00	1.00	1.00	1.00	1.00	1.00
Resident App use (Maximum)	16.00	3.00	33.00	5.00	3.00	19.00	21.00	1.00	6.00	7.00	4.00	26.00	3.00	4.00	5.00
Average App Usefulness	1.89	1.44	2.29	2.17	1.76	2.68	2.41	1.00	2.28	2.18	2.69	2.08	2.26	2.16	2.41
Standard Error App Usefulness	0.37	0.24	0.24	0.54	0.26	0.23	0.51	0.00	0.19	0.23	0.34	0.24	0.37	0.31	0.41

A. The percent of residents using the app is based on a total sample of 33 ALC residents.

B. The percent of residents using the app is based on a sample of 28 residents.
