# Supplementary figures and images for: Evaluating the Impact of Music & Memory’s Personalized Music and Tablet Engagement Program in Wisconsin Assisted Living Communities: Pilot Study
Source: JMIR Aging. 2019 Mar 14;2(1):e11599. doi: 10.2196/11599 (PMC6716484; doi:10.2196/11599)

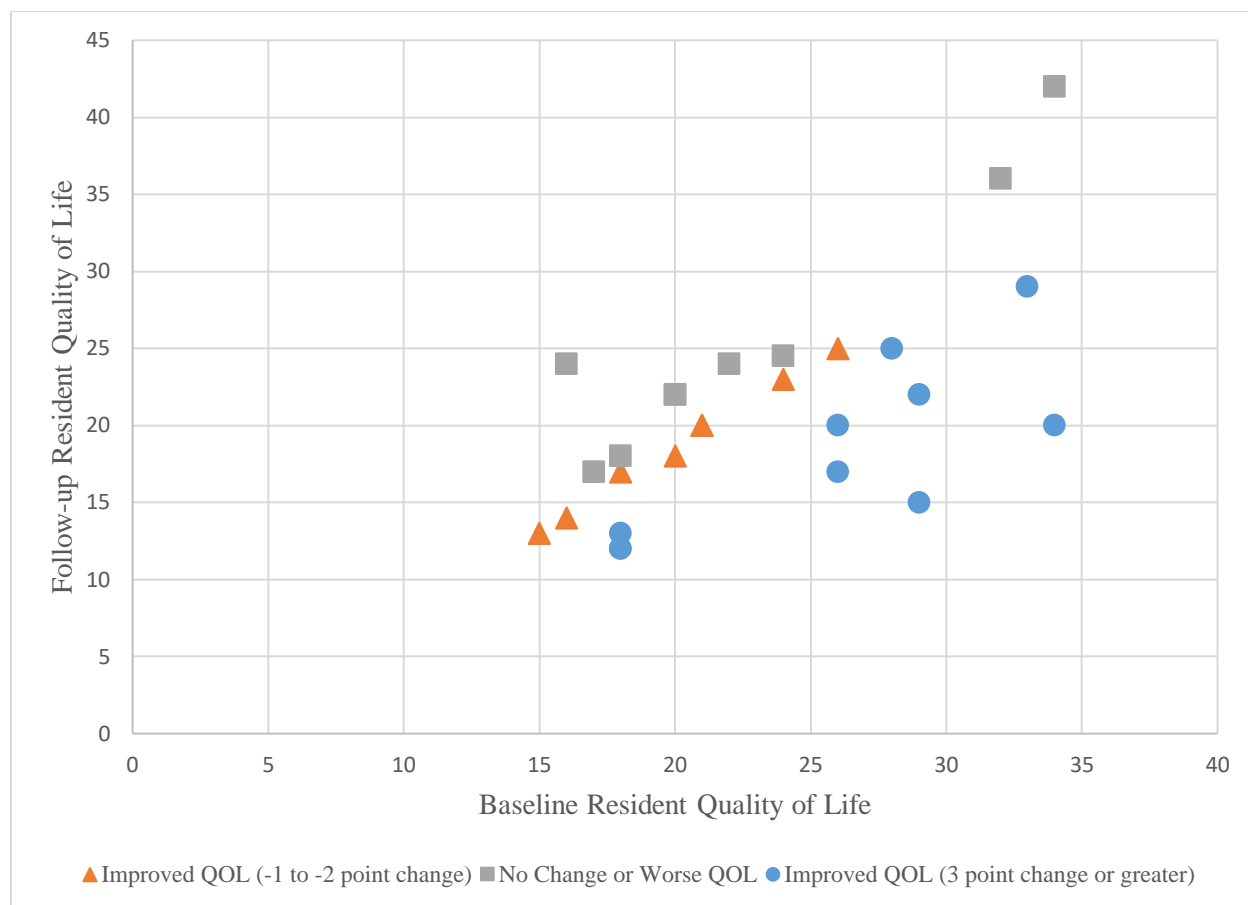

Supplement: Multimedia Appendix 7 [file aging_v2i1e11599_app7.pdf]
